# Individualised Ball Speed Prediction in Baseball Pitching Based on IMU Data

**DOI:** 10.3390/s21227442

**Published:** 2021-11-09

**Authors:** Larisa Gomaz, DirkJan Veeger, Erik van der Graaff, Bart van Trigt, Frank van der Meulen

**Affiliations:** 1 Delft Institute of Applied Mathematics, Delft University of Technology, 2628 CD Delft, The Netherlands; F.H.vanderMeulen@tudelft.nl; 2 BioMechanical Engineering, Faculty of Mechanical, Maritime and Materials Engineering, Delft University of Technology, 2628 CD Delft, The Netherlands; H.E.J.Veeger@tudelft.nl (D.V.); B.VanTrigt@tudelft.nl (B.v.T.); 3 Faculty of Behavioural and Movement Sciences, Vrije Universiteit Amsterdam, 1081 BT Amsterdam, The Netherlands; erik@pitchperfect-baseball.com

**Keywords:** ball velocity, inertial measurement unit, multilevel modeling, pitching, baseball

## Abstract

Ball velocity is considered an important performance measure in baseball pitching. Proper pitching mechanics play an important role in both maximising ball velocity and injury-free participation of baseball pitchers. However, an individual pitcher’s characteristics display individuality and may contribute to velocity imparted to the ball. The aim of this study is to predict ball velocity in baseball pitching, such that prediction is tailored to the individual pitcher, and to investigate the added value of the individuality to predictive performance. Twenty-five youth baseball pitchers, members of a national youth baseball team and six baseball academies in The Netherlands, performed ten baseball pitches with maximal effort. The angular velocity of pelvis and trunk were measured with IMU sensors placed on pelvis and sternum, while the ball velocity was measured with a radar gun. We develop three Bayesian regression models with different predictors which were subsequently evaluated based on predictive performance. We found that pitcher’s height adds value to ball velocity prediction based on body segment rotation. The developed method provides a feasible and affordable method for ball velocity prediction in baseball pitching.

## 1. Introduction

Proper pitching mechanics play an important role in both success and health of baseball pitchers. In overhead pitching, the lower extremity and trunk generate and transfer energy to the upper extremity. The optimal sequential activation of body parts while pitching, known as the kinetic chain, can result in reduced elbow and shoulder stress and maximise pitching performance [1,2]. On the other hand, poor mechanics can lead to increased loading of the elbow or shoulder, and increase the injury risk. Injuries of the throwing arm, such as the ones to the shoulder and elbow, are common in the overhead pitching motion of baseball. Major League Baseball pitchers are especially prone to injury because of the throwing velocities commonly seen approaching and even exceeding 100 mph. To create such high ball velocities, high energy levels pass through the components of the kinetic chain that affect the weakest links among them, especially the elbow [3]. Therefore, there is a need for assessment of the throwing technique that enables players to throw fast pitches in the strike zone without an overload.

Throwing velocity plays an important role in a success of a baseball game. Pitchers increase their chances for success by throwing faster and diminishing the hitter’s decision time of whether or not to strike the ball [4]. Furthermore, high ball velocities restrict the offense’s ability to advance bases and score runs [5]. Among other parameters, ball velocity is considered an important performance measure sought after by coaches and scouts. It enables baseball players to improve their ability to play and to be noticed by coaches and scouts for higher levels of competition. Therefore, every pitcher aims to increase the ball velocity [6,7].

The pitching biomechanics in baseball is studied to improve players’ performance and prevent sport-related injuries. With development of the measurement and analytical tools, pitching coaches and biomechanists can accurately analyse the rapid and complex movements during the pitching motion [8]. Although professional baseball teams have used biomechanical analysis for years already, recent advances in technology give amateur players and clubs opportunities to measure their mechanics and improve performance. Body worn sensors, such as inertial measurement units (IMUs), are a low-cost alternative to motion capture systems with passive markers, with no space limitation or cumbersome setup procedure. Portable, affordable, and easy-to-use, they monitor athlete’s performance without obstructing it [9].

As the quality of the throw is mainly determined by the pitcher’s throwing mechanics, we can use IMUs to measure kinematic parameters shown to be linked to ball velocity [7,8,10]. Enhancing pitching technique through the optimal position and timing of proper pitching mechanics, can result in a fast and accurate throw. Estimating ball velocity based on IMU recordings can be the first step towards assessment of the pitching technique that results in fast throws with reduced injury risk.

Ball velocity is mostly measured in high level games and in training situations. Although a radar gun gives an accurate reading of a ball velocity, a required strict protocol and high price represent a big issue for baseball clubs, especially the smaller ones. On the other hand, IMUs do not need a fixed location on the field for measuring ball velocity, thus they can be used on many different occasions. The previous studies demonstrated the potential use of IMUs for estimation of the ball velocity in different overhead-throwing sports, including baseball [11,12,13].

The use of IMUs represent a potential for the estimation of the ball speed in different on-field situations based on kinematic parameters measured by the same sensors. However, each pitcher is a unique individual and his individual characteristic may display individuality contributing to imparted velocity to the ball [7,14]. With IMUs, every throw of an individual pitcher can be recorded: during warm-up, training, before and during the game, which contributes to the element of individualisation. Therefore, in this paper, we present a method for predicting ball velocity in baseball pitching based on pitcher’s kinematics measured by IMUs and individual characteristics. We investigate the added value of the individuality to predictive performance of developed models.

## 2. Materials and Methods

### 2.1. Participants

Data were collected from 25 baseball pitchers with a mean age of 14.7 ± 1.5, mean body height 176.91 ± 11.03 cm and mean body weight 65.6 ± 14.4 kg. Participants were recruited from the national U18 baseball team, as well as all six baseball academies in The Netherlands, at which the most talented baseball players of that region train. This research was conducted in accordance with the Declaration of Helsinki and the Department of Human Movement Sciences’ local ethical committee approved the measurement protocol [ECB 2013-53]. Both participants and their parents were informed of the procedure and study aims before the start of the measurements. Informed consent was obtained from the parents of the participants before involvement in the study.

### 2.2. Methodology

The measurements were performed at the indoor facilities of the academies. After performing several anthropometric measurements, pitchers were given unlimited amount of time for their standard warm-up. They were instructed to prepare just as if they were going to pitch in a game. Warm-up included a general warm-up, j-band exercises, and longtoss, which is a standard warm-up for baseball pitchers before the game. The pitchers wore sneakers, athletic shorts, and no shirt. They also wore their catching glove to mimic the game situation as much as possible. After warm-up, the pitcher was instructed to perform 10 fastball pitches with maximal effort towards a catcher sitting behind home plate.

**Figure 1:**
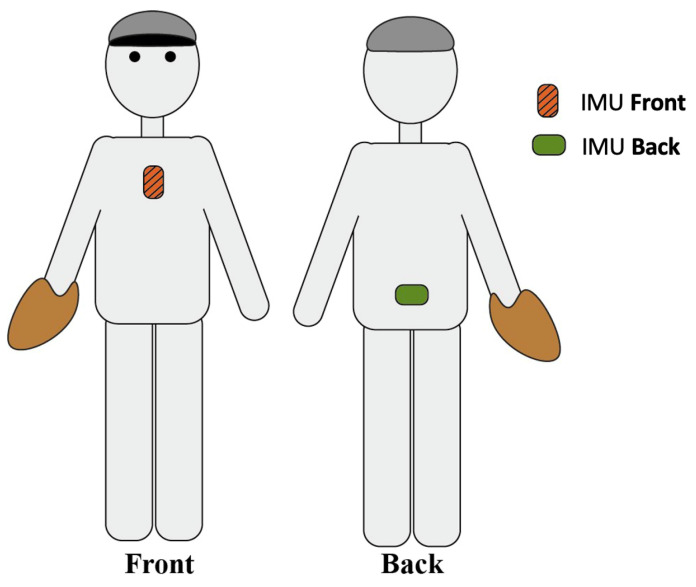
Placement of the sensors.

**Figure 2:**
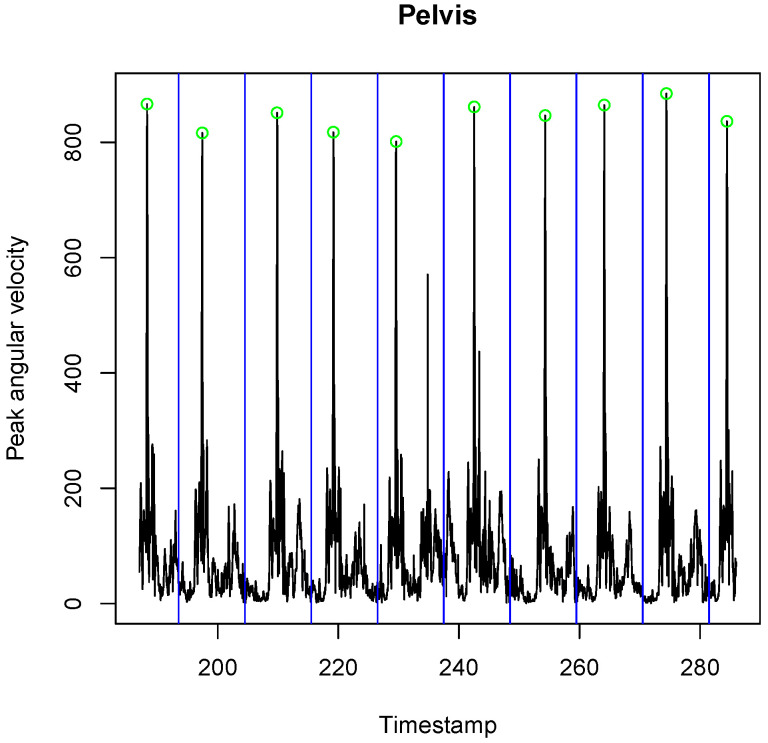
Segmenting the baseball pitches using gyroscope peaks. For the gyroscope signal, we calculated the peak angular velocity as its Euclidean norm. Each recording was manually segmented into parts containing only a single pitch. We performed the segmentation by plotting the entire gyroscope signal and locating the 10 peaks each corresponding to a pitch.

The pitching motion was recorded using two 9-DOF IMUs (MPU-9150, Invensense, San Jose, CA, USA, Accelerometer ± 16 g, Gyroscope ± 2000 deg/s). Sensors were rigidly attached to pelvis and sternum (Figure 1) using double-sided adhesive tape and used to record body segment rotation. Every sensor was embedded in a protective casing together with a battery and SD-card, onto which the data were logged at a sample frequency of 500 Hz. IMU’s gyroscope recorded angular velocities continuously throughout the participant’s session. Previous studies used peak values of kinematic measures to address their effect on the ball velocity in baseball pitching [2,6,7,10]. Therefore, for the gyroscope signal, we calculated the peak angular velocity as its Euclidean norm. Each recording was manually segmented into parts containing only a single pitch. We performed the segmentation by plotting the entire gyroscope signal and locating the 10 peaks each corresponding to a pitch (see Figure 2). This was done in a similar way in [11] for ball velocity data obtained in handball.

The ball velocity (mph) reached during the pitches was measured from behind the home plate using a Stalker pro 2 radar gun (Stalker Radar, Plano, TX, USA). We coupled recorded ball speed with corresponding peak angular velocities during single pitch.

### 2.3. Statistical Analysis

The repeated measurements of individual pitchers can be grouped into a hierarchical structure. The differences between participants arise from differences in personal characteristics, such as age, body weight, and height, that, next to the kinematic parameters of pitching mechanics, may contribute to increased ball velocity [1,2,8]. Observations in this study are ball throws nested within different participants and the link between individual- and group-level is participant’s indicator (ID).

Statistical models that can deal with units grouped on different levels are known as multilevel models. Multilevel models extend standard regression models to data which are structured in groups and where coefficients are allowed to vary by groups. The feature that distinguishes multilevel models from classical regression is the modeling of variation between groups. This enables us to study the effects that vary by group. Therefore, in this paper we introduce multilevel modeling as the main method for ball velocity prediction in baseball pitching.

**Figure 3:**
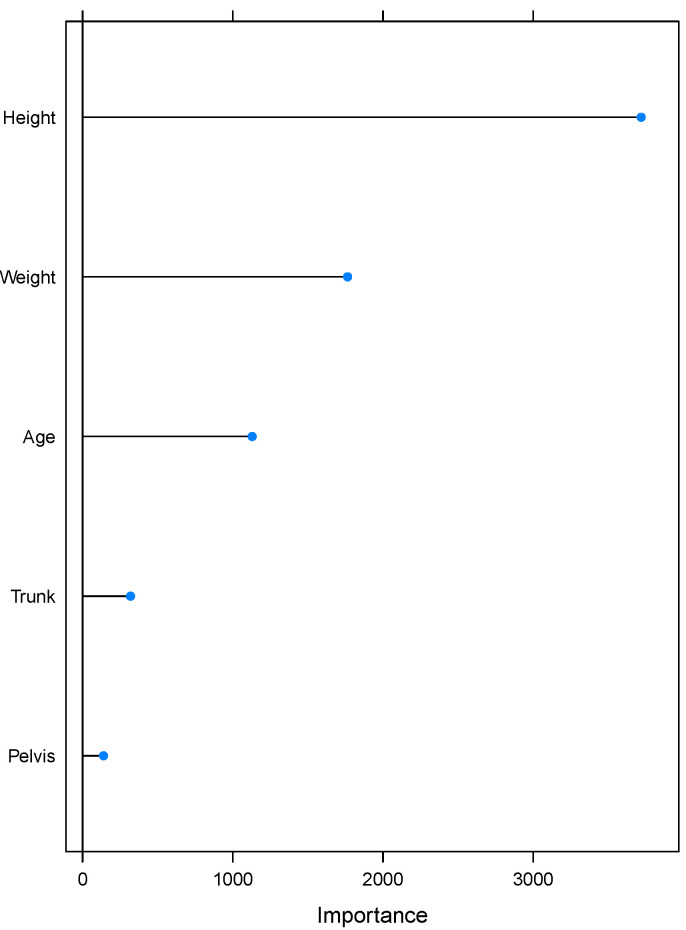
Visual representation of the variable importance calculated by applying random forest. The horizontal axis should be interpreted as a measure for relative importance of predictive variables. The figure reveals *Height* to be the most important predictor for ball speed which is, therefore, selected as group-level predictor.

At the same time as including repeated measurements of segment rotation per participant, the multilevel approach enables us to examine the added value of the individuality in ball velocity prediction. Group-level predictors were selected among personal characteristics that were collected prior to the measurements. We addressed the high correlation between pitcher’s height, weight and age. It is reasonable to expect that older pitchers will be taller and therefore weigh more. To select group-level predictors and avoid poor prediction performance due to correlation of predictors, we applied a random forest (see for instance chapter 8 in [15]), as implemented in the *caret* package [16]. Based on variable importance (Figure 3) calculated with *varImp* from a *caret* package, we selected pitcher’s height as a group-predictor. We developed three multilevel Bayesian regression models for ball velocity prediction using R 4.0.3 [17] and *rstanarm* [18,19].

In the following, $y_{i}$ denotes the ball speed for the observation indexed *i*.

1. Complete-pooling model (*Observations*)
  The complete-pooling model is a single classical regression model completely ignoring group information. In other words, the model treats all ball throws as different observations of the same participant. The model is given by
  (1) $$y_{i}=\beta_{0}+\beta_{1}x_{1i}+\beta_{2}x_{2i}+\epsilon_{i}$$
  where $\left\{x_{1i},x_{2i}\right\}$ are individual-level predictors, namely peak angular velocity of pelvis and trunk, respectively. The complete-pooling model does not make a distinction between different pitchers and in that way neglects their personal characteristics.
2. Two-level varying-intercept model (*Personal*)
  The two-level varying-intercept model is a regression that opposed to complete-pooling includes indicators for groups. In this model an intercept is calculated for every group and one joint slope is assumed for the entire sample. The model is given by
  (2) $$\begin{array}{cc} y_{i} & =\alpha_{j}+\epsilon_{i} \end{array}$$
  (3) $$\begin{array}{cc} \alpha_{j} & =\gamma_{0}+\gamma_{1}\bar{u_{j}}+\eta_{j} \end{array}$$
  where $\bar{u_{j}}$ is a centered group-level predictor, namely pitcher’s height. The group membership j[i] denotes pitcher *j* throwing a ball *i*. In this model pitching technique is neglected and the outcome depends only on height of an individual pitcher.
3. Two-level varying-intercept, varying-slope model (*Full*)
  The varying-intercept, varying-slope model represents the model in which both the intercept and the slope vary by group. The model is given by
  (4) $$\begin{array}{cc} y_{i} & =\alpha_{j[i]}+\beta_{1}x_{1i}+\beta_{2}x_{2i}+\epsilon_{i} \end{array}$$
  (5) $$\begin{array}{cc} \alpha_{j} & =\gamma_{0}+\gamma_{1}\bar{u_{j}}+\eta_{j} \end{array}$$
  and includes both individual- and group-level predictors. In both (3) and (5), the coefficient $\gamma_{0}$ can be interpreted as the ball speed of a ball thrown without any pelvis and trunk rotation by the pitcher of an average height. The $\epsilon_{i}$ in (1), (2) and (4) and $\eta_{j}$ in (3) and (5) represent independent error terms at each of the two levels.

All individual- and group-level predictors were rescaled to have sample variance 1. The scaling is done by dividing the centered predictor $\bar{u}$ by its standard deviation. We used scaling to transform the data to comparable values.

We used leave-one-out (LOO) cross-validation to select out of the three proposed models the model with best predictive performance. LOO resulted in a total of 224 folds as 224 pitches from 25 pitchers were included in the analysis. Following the approach in [20], the predictive performance of a model is defined as the expected log-predictive density (elpd). Predictive performance is a useful quantity in assessing a single model. It can be estimated by training the model on all observations except one and then predicting the hold-out observation. This is then repeated for all *n* observations

(6) $$\mathrm{elpd}=\sum_{i=1}^{n}\log p\left(y_{i}|y_{-i}\right)$$

where

(7) $$p\left(y_{i}|y_{-i}\right)=\int p\left(y_{i}|\theta \right)p\left(\theta |y_{-i}\right)d\theta$$

is the LOO predictive density upon leaving out the *i*th data point. If the posterior $p(\theta |y_{-i})$ is summarised by *B* simulation from $\theta^{i,b}$, then we can approximate $\log p(y_{i}|y_{i=1})$ by

$${\hat{\mathrm{elpd}}}_{i}=\frac{1}{B}\sum_{b=1}^{B}p\left(y_{i}|\theta^{i,b}\right)$$

leading to $\hat{\mathrm{elpd}}=\sum_{i=1}^{n}{\hat{\mathrm{elpd}}}_{i}$ as an estimate for $\hat{\mathrm{elpd}}$.

Different models can be compared against each other according to their elpd-value. Suppose we wish to compare models $M_{1}$ and $M_{2}$, with estimated elpd values ${\hat{\mathrm{elpd}}}^{1}$ and ${\hat{\mathrm{elpd}}}^{2}$, respectively.

Since the same set of *n* data points is being used to fit all models, we can use a paired estimate to compute a standard error of their difference:

(8) $$\mathrm{se}\left({\hat{\mathrm{elpd}}}^{1}-{\hat{\mathrm{elpd}}}^{2}\right)=\sqrt{nV_{i=1}^{n}\left({\hat{\mathrm{elpd}}}_{i}^{1}-{\hat{\mathrm{elpd}}}_{i}^{2}\right)}.$$

Here, for numbers ${\left\{a_{i}\right\}}_{i=1}^{n}$ we define $V_{i=1}^{n}a_{i}=\frac{1}{n-1}\sum_{i=1}^{n}{\left(a_{i}-{\bar{a}}_{n}\right)}^{2}$.

## 3. Results

We included in the analysis 224 pitches from 25 pitchers for which the ball velocity was recorded and sensor clipping did not occur. Characteristics of the measured ball and peak angular velocities of pelvis and trunk are summarised in Table 1.

**Figure 4:**
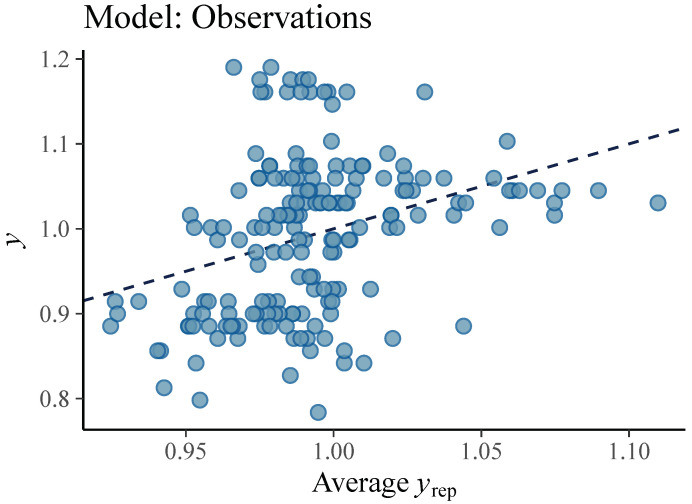
Ball velocity observations (dots) vs. average simulated value of the ball speed (line) from the posterior predictive distribution of the *Observations* model. This graphical representation suggests that *Observations* model leaves a large amount of variation in the data unexplained.

**Figure 5:**
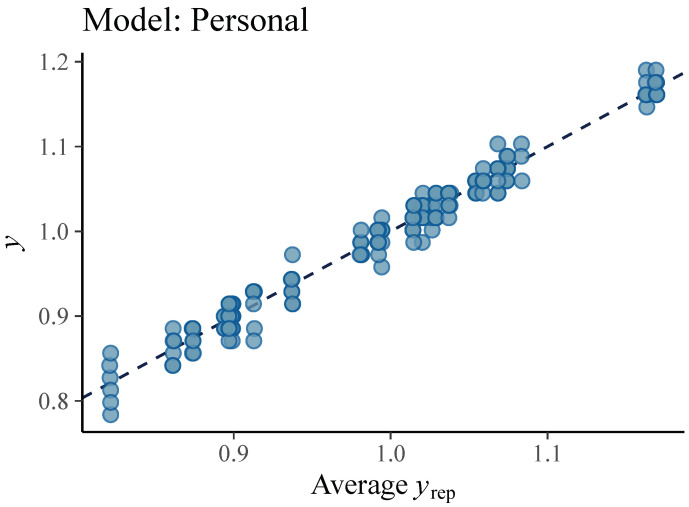
Ball velocity observations (dots) vs. average simulated value of the ball velocity (line) from the posterior predictive distribution of the *Personal* model. This graphical representation suggests that *Personal* model is a good fit to collected data.

**Figure 6:**
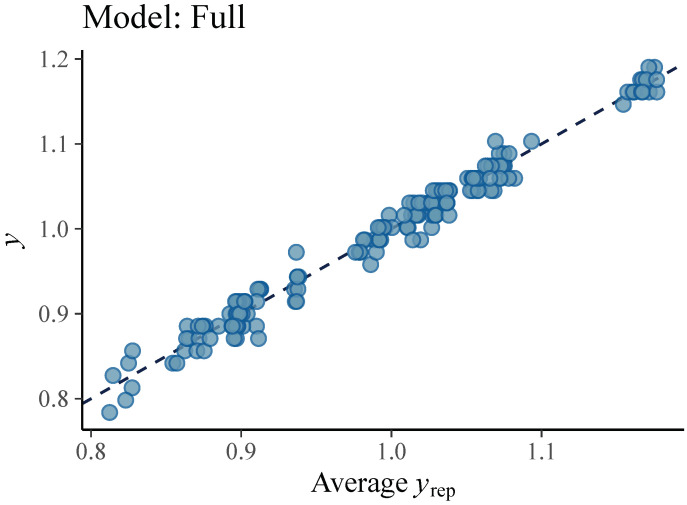
Ball velocity observations (dots) vs. average simulated value of the ball velocity (line) from the posterior predictive distribution of the *Full* model. This graphical representation suggests that *Full* model is a good fit to collected data.

We consider the model called *Observations* model as base model. The other two proposed models, *Personal* and *Full*, are extensions since they have two instead of one level and they introduce the group participation that makes a distinction between pitchers of a different height. Therefore, comparing the developed models determines the contribution of the kinematic parameters related to pitching mechanics and body height of a pitcher to accuracy of ball speed prediction. The graphical representations (Figure 4, Figure 5 and Figure 6) show that both the models *Personal* and *Full* provide a good fit to the observed data, while the fit of *Observations* is unsatisfactory.

The *Full* model is a preferable model, followed by the *Personal* and *Observations* model (see Table 2 and Table 3).

## 4. Discussion

The aim of this study was to predict a ball velocity in baseball pitching such that prediction is tailored to the individual pitcher. The proposed method included pitcher’s body segment rotation, which determines his technique, and pitcher’s height that displays individuality in imparted velocity to a ball. We used multilevel modeling to develop three models with different predictors and examined their predictive performance. By comparing developed models, we investigated the added value of individuality to ball velocity prediction.

Ball velocities presented in this study are similar to the ones reported in the previous studies. Pitchers with a mean age of 14.7 ± 1.5 years threw balls with average velocity 30.6 ± 2.9 m/s, while Dun [21] reported average ball velocity of 26.3 ± 3.8 m/s measured in a population of youth pitchers throwing fastballs.

In the overhead pitching, the lower extremity and trunk generate and transfer energy to the upper extremity. To examine the relationship between ball velocity and variations in pitching biomechanics on individual level, previous studies identified maximum pelvis and trunk angular velocity as kinematic parameters linked to ball velocity [7,8,10]. Recent technological developments brought IMUs to a spotlight as an alternative to marker-based systems used in a laboratory setting. Since IMU’s gyroscope enables measuring body segment rotation, we assessed pitching technique by positioning IMU sensors on pelvis and trunk. Measured peak angular velocity of pelvis of 690.2 ± 90.9 deg/s and trunk of 1172.4 ± 239.5 deg/s supports the findings in previous studies [2,21]. As the gyroscopes recorded angular velocities continuously throughout the participant’s session, manual segmentation was required. In future work, we wish to develop a method for automatic detection of single throws and segmentation of the continuous-time gyroscope signal when the boundaries between different throws are unclear. This will automatise the use of predictive models for predicting ball velocity. Filtering methods from signal processing may prove to be useful for this purpose.

Among the compared models, model *Full* shows the best predictive performance (Table 3). Model *Observations* is worse than model *Full* by 308.3 of log predictive probability values. The difference in estimated elpd-values is big compared to estimated standard error of 13.5. Hence, adding pitcher’s height to the *Observations* model improves predictive accuracy. Model *Personal* includes only the pitcher’s height as a predictor and ignores the pitching technique. The model shows that taller pitchers throw faster and it is possible to already estimate ball velocity only by knowing the pitcher’s height. This information can be useful for scouts in search for baseball talents. A pitcher’s height compared to other personal characteristics, such as age and weight, is the most important predictor for ball velocity in baseball pitching. On the other hand, of course neither pitchers nor coaches can influence height. The outcome of this paper demonstrates the added value of a pitcher’s height to predictive accuracy.

The proposed method can potentially be adopted in baseball practice. IMUs are easy-to-wear low cost sensors that do not influence a pitcher’s performance and can be a valuable source of data. It can provide information on pitching performance in every situation and with a method proposed in this paper, gain ball velocity without use of a radar gun. Ball velocity prediction can give a better insight into pitcher’s performance and represents a potential for predictions of future throwing speed when pitchers grow taller.

For future studies, we suggest also to include separation time and pitch types in the presented model. Following the concept of a kinetic chain, the relative timing of the moments of pelvis and trunk peak angular velocity, when throwing fastballs, is associated with ball velocity in skilled pitchers [22]. Furthermore, to the best of our knowledge, no study has classified pitch types based on IMU data solely. Classification of pitch types outside the laboratory or game environment provides benefits in designing and outlining training routines and represents a potential research direction in the future. Following the segmentation of continuous gyroscope signals, we suggest extracting additional features next to the peak angular velocities, such as skewness, mean, and difference between minimum and maximum. This would result in more parameters that may be included in the model and improve the classification of different pitch types and the prediction of ball velocity.

## Figures and Tables

**Table 1:** Summary of measured ball and peak angular velocities.

	Mean ± Standard Deviation
Peak pelvis angular velocity (deg/s)	690.2 ± 90.9
Peak trunk angular velocity (deg/s)	1172.4 ± 239.5
Ball velocity (mph)	68.3 ± 6.5

**Table 2:** Comparison of fitted models. The rows show the difference in $\hat{\mathrm{elpd}}$, with estimated standard error in brackets, between the *Full* model and remaining models (*Personal* and *Observations*).

${\hat{\mathrm{elpd}}}^{\mathrm{Full}}-{\hat{\mathrm{elpd}}}^{\mathrm{Personal}}$	−5.5 (3.3)
${\hat{\mathrm{elpd}}}^{\mathrm{Full}}-{\hat{\mathrm{elpd}}}^{\mathrm{Observations}}$	−308.3 (13.5)

**Table 3:** Comparison of fitted models.

	$R^{2}$	RMSE
Full	0.975	0.014
Personal	0.973	0.014
Observations	0.137	0.089

## Data Availability

The data presented in this study are openly available in 4TU.ResearchData at https://doi.org/10.4121/16691947 (accessed on 1 November 2021).

## References

[1] Pappas A.M., Zawacki R.M., Sullivan T.J. (1985). Biomechanics of baseball pitching. A preliminary report. Am. J. Sport. Med..

[2] Seroyer S.T., Nho S.J., Bach B.R., Bush-Joseph C.A., Nicholson G.P., Romeo A.A. (2010). The kinetic chain in overhand pitching: Its potential role for performance enhancement and injury prevention. Sports Health.

[3] Anz A.W., Bushnell B.D., Griffin L.P., Noonan T.J., Torry M.R., Hawkins R.J. (2010). Correlation of Torque and Elbow Injury in Professional Baseball Pitchers. Am. J. Sport. Med..

[4] Hay J.G. (1993). The Biomechanics of Sports Techniques.

[5] Lehman G., Drinkwater E.J., Behm D.G. (2013). Correlation of throwing velocity to the results of lower-body field tests in male college baseball players. J. Strength Cond. Res..

[6] Fortenbaugh D., Fleisig G.S., Andrews J.R. (2009). Baseball Pitching Biomechanics in Relation to Injury Risk and Performance. Sports Health.

[7] Werner S.L., Suri M., Guido J.A., Meister K., Jones D.G. (2008). Relationships between ball velocity and throwing mechanics in collegiate baseball pitchers. J. Shoulder Elbow Surg..

[8] Dowling B., Pearl C., Laughlin W., Fleisig G. Relationship of pelvis and trunk kinematics to ball velocity in professional baseball pitchers. Proceedings of the 40th American Society of Biomechanics (ASB) Annual Meeting (ASB 2016).

[9] Camomilla V., Bergamini E., Fantozzi S., Vannozzi G. (2018). Trends Supporting the In-Field Use of Wearable Inertial Sensors for Sport Performance Evaluation: A Systematic Review. Sensors.

[10] Van der Graaff E., Hoozemans M.J.M., Nijhoff M., Davidson M., Hoezen M., Veeger H.E.J. (2018). Timing of peak pelvis and thorax rotation velocity in baseball pitching. J. Phys. Fit. Sport. Med..

[11] Skejø S.D., Bencke J., Møller M., Sørensen H. (2020). Estimating Throwing Speed in Handball Using a Wearable Device. Sensors.

[12] McGrath J., Neville J., Stewart T., Clinning H., Cronin J. (2021). Can an inertial measurement unit (IMU) in combination with machine learning measure fast bowling speed and perceived intensity in cricket?. J. Sport. Sci..

[13] Gençoğlu C., Gümüş H. (2020). Standing handball throwing velocity estimation with a single wrist-mounted inertial sensor. Ann. Appl. Sport Sci..

[14] Matsuo T., Escamilla R.F., Fleisig G.S., Barrentine S.W., Andrews J.R. (2001). Comparison of Kinematic and Temporal Parameters between Different Pitch Velocity Groups. J. Appl. Biomech..

[15] Kuhn M., Johnson K. (2013). Applied Predictive Modeling.

[16] Kuhn M. (2020). Caret: Classification and Regression Training. R Package Version 6.0-86. https://CRAN.R-project.org/package=caret.

[17] R Core Team (2020). R: A Language and Environment for Statistical Computing.

[18] Goodrich B., Gabry J., Ali I., Brilleman S. (2020). Rstanarm: Bayesian Applied Regression Modeling via Stan. R Package Version 2.21.1. https://mc-stan.org/rstanarm.

[19] Muth C., Oravecz Z., Gabry J. (2018). User-friendly Bayesian regression modeling: A tutorial with rstanarm and shinystan. Quant. Methods Psychol..

[20] Vehtari A., Gelman A., Gabry J. (2017). Practical Bayesian model evaluation using leave-one-out cross-validation and WAIC. Stat. Comput..

[21] Dun S., Loftice J., Fleisig G.S., Kingsley D., Andrews J.R. (2008). A biomechanical comparison of youth baseball pitches: Is the curveball potentially harmful?. Am. J. Sport. Med..

[22] Van der Graaff E., Hoozemans M., Nijhoff M., Davidson M., Hoezen M., Veeger D. The role of pelvis and thorax rotation velocity in baseball pitching. Proceedings of the 34th International Conference on Biomechanics in Sports.

